# AnaConDa™ and Mirus™ for intensive care sedation, 24 h desflurane versus isoflurane in one patient

**DOI:** 10.1186/s40064-016-2065-0

**Published:** 2016-04-06

**Authors:** Hagen Bomberg, Heinrich V. Groesdonk, Martin Bellgardt, Thomas Volk, Andreas Meiser

**Affiliations:** Department of Anaesthesiology, Intensive Care Medicine and Pain Medicine, Saarland University Medical Center, Kirrbergerstrasse 1, 66421 Homburg/Saar, Germany; Department of Anaesthesiology and Intensive Care Medicine, St. Josef Hospital, Katholisches Klinikum Bochum, Ruhr-University, Bochum, Germany

**Keywords:** Desflurane, Sedation, Intensive care unit (ICU), Mirus™, AnaConDa™

## Abstract

**Introduction:**

With the AnaConDa™ system, inhalational sedation in the intensive care unit has become popular. The device can be used with common intensive care unit ventilators and is inserted between the Y-piece and the patient. Liquid isoflurane or sevoflurane are delivered by a syringe pump. 90 % of anesthetic exhaled by the patient is absorbed by a reflector and resupplied during the next inspiration. The new Mirus™ system also uses a reflector. Its control unit identifies end-tidal concentrations from the flow, injects anesthetics during early inspiration, controls anesthetic concentrations automatically, and can also apply desflurane. The AnaConDa™ and Mirus™ system are certified ‘conformité établi’, however, little is known about the Mirus™ and case reports are still lacking.

**Case description:**

We used the Mirus™ with desflurane for 24 h in a patient suffering from acute respiratory distress syndrome. The patient was treated with kinetic lateral rotational therapy. While deeply sedated, our patient breathed 9.0–12.0 l min^−1^ spontaneously. Thereafter, awakening and wash-out were considerably shorter than after isoflurane in the same patient with AnaConDa™. There were no major problems during the sedation. However, consumption of desflurane was high.

**Conclusion:**

Desflurane sedation with the Mirus™ seems promising, but the reflector should be improved to absorb and resupply more of the anesthetic agent.

## Introduction

Inhalational sedation in the setting of an intensive care unit (ICU) has become popular with the arrival of the AnaConDa™ system (Sedana Medical, Uppsala, Sweden) (Sackey et al. 2004). Instead of a circle system, the AnaConDa™ uses a specific anesthetic reflector to save on the anesthetic agent. A syringe pump delivers liquid isoflurane or sevoflurane into a porous hollow rod called “evaporator” (Meiser et al. 2009). No vaporizer and no fresh gas flow to carry the anesthetic to the patient are needed. The AnaConDa™ can be used with common intensive care ventilators. The use of anesthesia ventilators with circle systems in the intensive care setting is not an option as they are not licensed for stand-alone use and poorly support spontaneous breathing.

The German sedation guidelines recommend inhalational sedation in patients ventilated via tracheal tube or tracheostomy as an alternative to intravenous sedation (Baron et al. 2015).

A reflection system with desflurane would be advantageous because of its more favorable kinetics (Bennett et al. 1992; Tsai et al. 1992) and higher stability compared to sevoflurane (Perbet et al. 2013; Rohm et al. 2009). Desflurane cannot be applied via syringe pump as in the AnaConDa™ because of its low boiling point. Desflurane needs a special system for vaporization and administration.

The new Mirus™ system (Pall Medical, Dreieich, Germany) is the first to use desflurane with anesthetic reflection instead of a circle system. The new Mirus™ as well as the AnaConDa™ are certified ‘conformité établi’ (CE) for the use in the intensive care unit (ICU). Nevertheless, there are no existing case reports about the Mirus™; in contrast, the AnaConDa™ is well known. Therefore, we evaluated the functioning of the Mirus™ in a critically ill patient and compared wash-out of desflurane administered with the Mirus™ with that of isoflurane administered with the AnaConDa™ in the same patient.

## Case description

A 70-year-old woman, 162 cm tall, weighing 105 kg, suffered from paralytic ileus after surgical removal of an abdominal mass. Pathology revealed a follicular non-Hodgkin lymphoma grade 1–2. After severe aspiration, she was admitted to our ward. She was stabilized and her trachea was intubated; bronchoscopy confirmed severe aspiration of gastric contents. The patient developed moderate acute respiratory distress syndrome and was treated with kinetic lateral rotational therapy (Rotorest™ bed, KCI, San Antonio, Texas, USA).

**Fig. 1:**
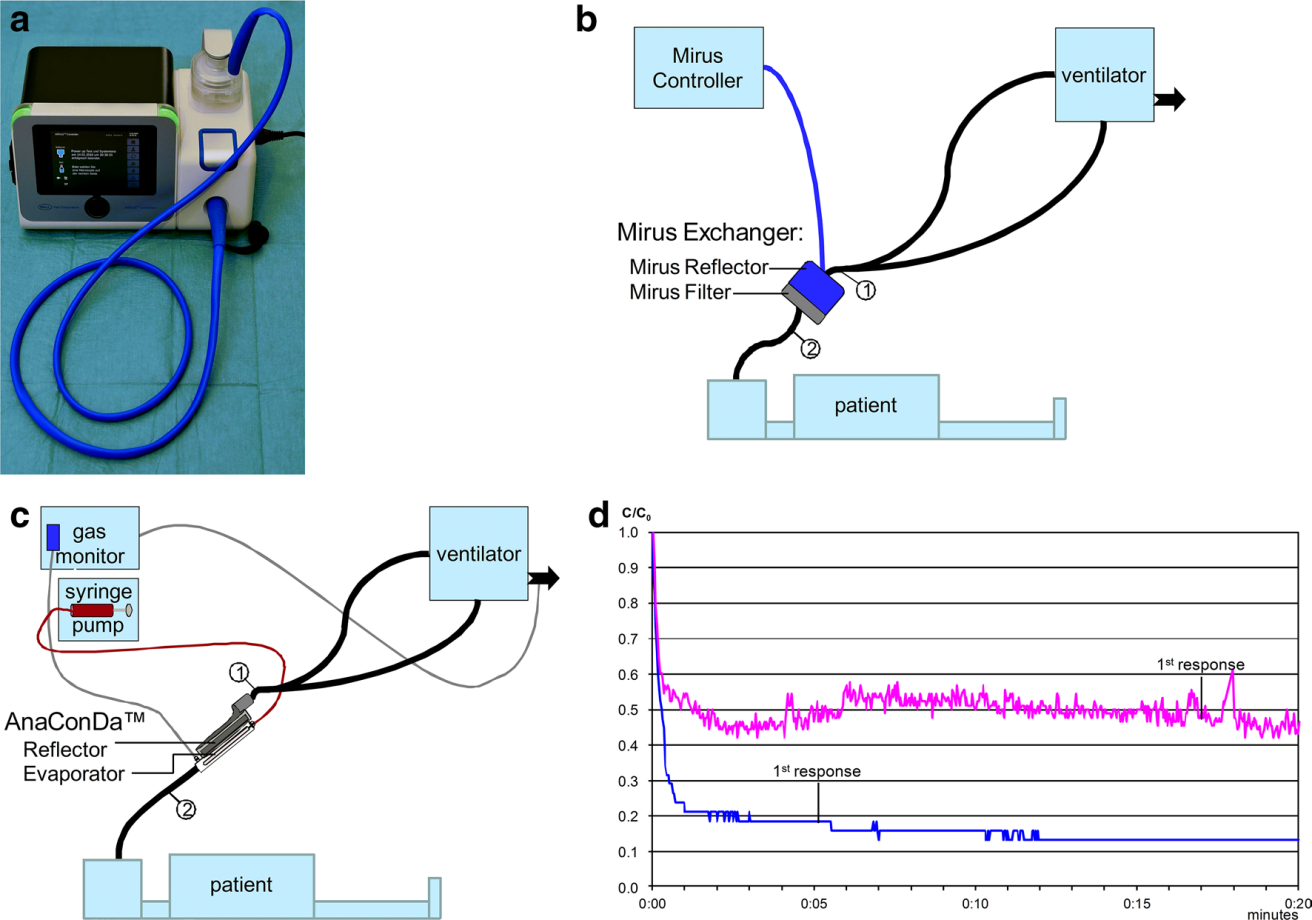
**a** Mirus™ device. **b** Setup of the Mirus™ system: the interface (“Mirus™ Exchanger”) is inserted between the Y-piece (*1*) and the endotracheal tube (*2*). The interface is connected with the control unit by a multi-lumen cable (*blue line*). An active anaesthesia gas scavenging system (*black arrow*) connected to the vacuum system is provided by the manufacturer. **c** Setup of the AnaConDa™: the AnaConDa™ is inserted between the Y-piece (*1*) and the endotracheal tube (*2*). Liquid isoflurane or sevoflurane is administered by a syringe pump into a hollow rod (“Evaporator”). An external gas monitor must be used. Active and passive scavenging systems are available and should be connected to the gas outlet of the ventilator (*black arrow*). Sample gas should also be scavenged. **d** Wash-out kinetics of desflurane (*blue*) and isoflurane (*purple*) over 20 min in the same patient after 24-h application. The end-tidal desflurane concentration decreases down to 20 % of the initial concentration (C_0_; 3.8 vol%) after 1 min; the isoflurane concentration only decreases to 50 % of C_0_ (0.8 vol%) during the whole observation period. The patient first responded after 5.08 min at 0.7 vol% desflurane and after 17 min at 0.44 vol% isoflurane

After approval of the ethics committee (Landesärztekammer des Saarlandes; reference 199/09) and informed consent by the legal representative, later confirmed by the patient herself, sedation was switched on day four from midazolam/sufentanil to desflurane, 3.3–3.8 vol%, administered with the Mirus™ for 24 h (Fig. 1a). Sufentanil was decreased from 0.95 to 0.38 µg kg^−1^ h^−1^.

A desflurane specific version of the Mirus™ was connected to an Evita 4 ICU ventilator as prescribed by the manufacturer (Fig. 1b). The Mirus™ interface, inserted between the Y-piece and the endotracheal tube, consists of two parts: the “Mirus Reflector”, which comprises an anesthetic reflector and endings of cables for direct gas injection, for measuring pressure, flow, and gas concentrations; and the “Mirus Filter” for conditioning of breathing gas (heat moisture exchanger) and removal of bacteria, viruses, and particles.

The control unit displays respiratory pressure and flow continuously. The end-tidal desflurane concentration is controlled automatically to a set target value. The speed of control can be chosen as slow (symbol: tortoise), moderate (symbol: hare), or high (symbol: cheetah), leading to higher inspiratory concentrations during wash-in. We selected moderate speed of control.

Thereafter, isoflurane, 0.5–0.7 vol%, was applied with the AnaConDa™ and the Vamos gas monitor. Setup was according to the manufacturer’s instructions (Fig. 1c). Liquid isoflurane was delivered by a syringe pump (Perfusor fm; Braun Melsungen AG, Germany). The same Evita 4 ICU ventilator and the same ventilator accessories were used.

End-tidal concentrations, the bispectral index (BIS™, Aspect Medical Systems, Newton, MA, USA) and the Richmond Agitation and Sedation Scale (RASS) score were recorded manually once every hour.

During gas application with both systems, RASS scores were between -4 and -5 and the bispectral index between 35 and 55. While deeply sedated, our patient breathed 9.0–12.0 l min^−1^ spontaneously. There were no major problems during the sedation, no relevant increase in arterial carbon dioxide tension, and no relevant changes in hemodynamics. During 24-h sedation with the Mirus™, 1261 ml desflurane were consumed as opposed to 96 ml isoflurane per 24 h with the AnaConDa™.

In two sedation windows, after 24 h desflurane and 24 h isoflurane respectively, wash-out of both gases was recorded with the Vamos gas monitor. The Mirus™ interface and the AnaConDa™ respectively were replaced by a heat moisture exchanger with a gas sampling port (Humid-Vent Filter Compact S, Teleflex Medical GmbH, Kernen, Germany). Sedation recovery was monitored by an observer calling the patient’s name every 30 s.

Wash-out of desflurane after 24 h was considerably shorter than that of isoflurane. With the Mirus™, we observed the patient’s first response 5.08 min after stopping desflurane at an end-tidal concentration of 0.7 vol% as opposed to 17 min after stopping isoflurane at a concentration of 0.4 vol% using the AnaConDa™ system (Fig. 1d).

Oxygenation improved under lateral rotational therapy (from day 4 until day 12 after ICU admission). Until day 12, the patient was sedated with the AnaConDa™ (Isoflurane), and thereafter with sufentanil and bolus doses of midazolam. Invasive ventilation was continued intermittently until day 29; the patient was transferred to the ward on day 68 and discharged to a rehabilitation center on day 87.

## Discussion

Overall, sedation of our patient with desflurane and the Mirus™ during 24 h was completely satisfactory. Using a low opioid dose, spontaneous breathing was possible in a patient deeply sedated to avoid unpleasant perceptions during kinetic lateral rotational therapy. In comparison with isoflurane and the AnaConDa™, we observed a comparable sedation quality, however, wash-out of desflurane and awakening were considerably shorter. Still, the Mirus™ reflector should be improved to save more anesthetics.

In view of the better kinetics of desflurane with known shorter wash-out time (Juvin et al. 1997; Bailey 1997), this is not surprising. However, this is the first report on wash-out of desflurane and isoflurane after such a long exposure time, starting from rather low concentrations in a critically ill patient. As the patient served as her own control, and the difference may be expected from the kinetics, our results are reliable and underline the magnitude of the difference: not only was the time to first response 5 min versus seventeen but isoflurane concentration quickly declined to about 50 % of the initial concentration and then remained stable because of redistribution from poorly perfused tissues. Desflurane concentration declined to 20 % of the initial concentration and continued to decline thereafter, which implies that there was very little accumulation. This should be considered an advantage in itself, as the patient will not only respond to verbal commands but regain higher cognitive functions far quicker than after isoflurane.

Kinetic lateral rotational therapy needs deep sedation to avoid unpleasant perceptions. However, deep intravenous sedation is associated with severe respiratory depression necessitating mechanical ventilation. Patients suffering from acute respiratory distress syndrome benefit from spontaneous breathing. Using isoflurane or desflurane and a low opioid dose, spontaneous breathing is possible in a patient deeply sedated. Moreover, long-term deep intravenous sedation has been associated with an increased mortality (Shehabi et al. 2013). In contrast, long-term isoflurane sedation seems to avoid this increase in mortality (Bellgardt et al. 2016).

With the AnaConDa™, an external gas monitor must be used. This monitor will erroneously display early inspiratory peak concentrations as end-tidal. This phenomenon has been described by our group (Meiser and Laubenthal 2005). In contrast, the Mirus™ comprises its own monitor for gas concentration, pressure, and flow measurements. Unlike other monitors, the Mirus™ assigns the concentration measurements to the phases of the respiratory cycle not depending on carbon dioxide but on the flow. Obviously, the time lag associated with side stream gas measurements must be taken into account.

In our previous bench study, we found a high accuracy and precision of the end-tidal concentrations displayed by the Mirus™ (Bomberg et al. 2014). However, there was a swing of 20 % around the target concentration with a periodicity of 2.6 min which may be criticized. In our patient, we did not see oscillations in the bispectral index or any clinical signs of an alternating sedation level.

In our previous bench study, mean inspiratory concentrations during desflurane injections were only 134 % (SD 12 %) of the preceding end-tidal concentration (Bomberg et al. 2014). This is unlikely to elicit sympathetic activation as described in the literature (Ebert et al. 1998; Weiskopf et al. 1994). In our patient, we did not notice sympathetic activation during washing-in of desflurane.

However, consumption of desflurane was high. Also, with the AnaConDa™, consumption of isoflurane and sevoflurane was very high in the beginning (Enlund et al. 2001). With technical modifications of the reflector, this could be improved (Meiser et al. 2009).

For 24-h sedation with 3.5 vol% desflurane on average, 1261 ml desflurane were consumed. In our previous bench study (Bomberg et al. 2014), by a simple modification of the Mirus™ anaesthetic reflector, we could cut consumption in half. Given a six times higher potency of isoflurane, 600 ml of desflurane consumed would seem reasonable compared to 100 ml isoflurane.

During the application, only two minor problems occurred. First, filling of the desflurane reservoir with a capacity of 250 ml took more than 120 s. Secondly, the volume expansion by injection of desflurane repeatedly triggered alarms of the ventilator because the ventilator measured a higher expired than inspired tidal volume. Deactivation of the flow sensor may be admissible as the Mirus™ also measures and displays respiratory parameters. However, staff must be instructed about this.

Our favorable result is in line with two older reports on desflurane sedation (Sharpe et al. 2002; Meiser et al. 2003). Desflurane sedation with the Mirus™ system should be tested in randomized controlled trials focusing on feasibility, sedation control, and outcome parameters.

## Conclusion

Our findings imply that Mirus™ is a useful, unique system for administration of desflurane with common intensive care ventilators. Spontaneous breathing is possible in a patient deeply sedated, and awakening after desflurane is considerably shorter compared to isoflurane.
